# Amitriptyline overdose—an uncommon cause of acute transient exotropia presenting to the emergency setting: a case report

**DOI:** 10.1186/s13256-023-04057-y

**Published:** 2023-09-08

**Authors:** Arun Rajaratnam

**Affiliations:** National Hospital Kandy, Kandy, Sri Lanka

**Keywords:** Amitriptyline poisoning, Transient exotropia, Resource-poor setting

## Abstract

**Background:**

Acute presentations of acquired exotropia or divergent alignment of either or both eyes are commonly observed following intracranial vascular events, trauma, orbital, and endoscopic sinus surgeries.

**Case presentation:**

The reported case is about a 16-year-old previously healthy Tamil female who presented to the emergency department with a few hours of reduced responsiveness. With the only clue in the history being about a family conflict the previous day, the examination revealed a noticeable exotropia along with a constellation of anticholinergic findings: a low Glasgow Coma Score, mydriasis, tachycardia, floppy limbs, exaggerated reflexes, and a palpable urinary bladder. Amitriptyline overdose leading to significant neurological involvement was suspected, and she was immediately offered urine alkalinization. Resources for urine and blood toxicological studies were not available. The patient gained consciousness 24 hours later and confirmed an overdose of ten amitriptyline tablets. Exotropia, a unique manifestation of this patient’s neurotoxicity, spontaneously resolved in 36 hours.

**Discussion and conclusions:**

The reported case is about an uncommon clinical finding of exotropia seen in a common toxicological emergency: acute amitriptyline overdose. The importance of having a wide knowledge of various clinical presentations of drug toxicities is highlighted here, as any delay in diagnosis or initiation of life-saving measures could have resulted in fatal consequences.

## Background

Overdose with tricyclic antidepressants (TCAs) such as amitriptyline is a frequent presentation in the emergency setting and can culminate in fatal consequences if not identified and treated appropriately. A probable diagnosis of TCA overdose is supported by the presence of an anticholinergic toxidrome that includes tachycardia, urinary retention, mydriasis, hyperreflexia, and a positive Babinski, along with metabolic acidosis and characteristic electrocardiographic (ECG) changes. Identification and interpretation of the constellation of clinical features are crucial in making the diagnosis and promptly initiating life-saving measures, particularly when a clear history of overdose is not present and when adequate resources for toxicological analysis of blood or urine are unavailable. The reported case is about a young girl initially presenting with unconsciousness, and the examination revealed a low Glasgow Coma Scale, anticholinergic toxidrome features, and a unique, unmistakable presence of exotropia (divergence of one or both eyes). Acute amitriptyline poisoning with significant neurological involvement was considered and promptly managed with urine alkalinization. With no facilities for toxicological studies available, the diagnosis of amitriptyline overdose was confirmed only 24 hours later by the patient herself. The reported case and clinical images are mainly aimed at creating awareness of exotropia as an uncommon neuro-ophthalmologic finding in a commonly encountered toxicological emergency. The case also highlights the importance of being aware of the various clinical presentations to avoid delays in diagnosis or the initiation of life-saving measures.

## Case presentation

A 16-year-old previously well Tamil girl was brought to the emergency department by her elder sister, in an unarousable state for about 2–3 hours. The sister disclosed that the patient was found unconscious in their home garden. The exact time of onset and the nature of the symptoms were unknown. Apart from the history of a recent argument with her parents, there was nothing else significant. There was no preceding history of febrile illness, headache, seizure disorder, or major gastrointestinal, urinary, or menstrual disturbances. Her last period of menstruation was unknown. There were no previous medical or surgical comorbidities, and she was not on any long-term medications. The accompanying relative revealed that the patient’s mother was on certain medications for several diseases, including diabetes, but the exact details of the medications were unknown. There were no suicidal notes or empty pill boxes at the scene. The patient had not been acting suspiciously or expressing any ideas of self-harm. There was no history of alcohol intake, smoking, or use of illicit substances. Given the acute presentation of unconsciousness, central nervous system ischemic or hemorrhagic vascular events, acute metabolic encephalopathies, medication overdose, plant poison ingestion, and even snake bites with neurological envenomation features were considered as top differentials.

**Fig. 1:**
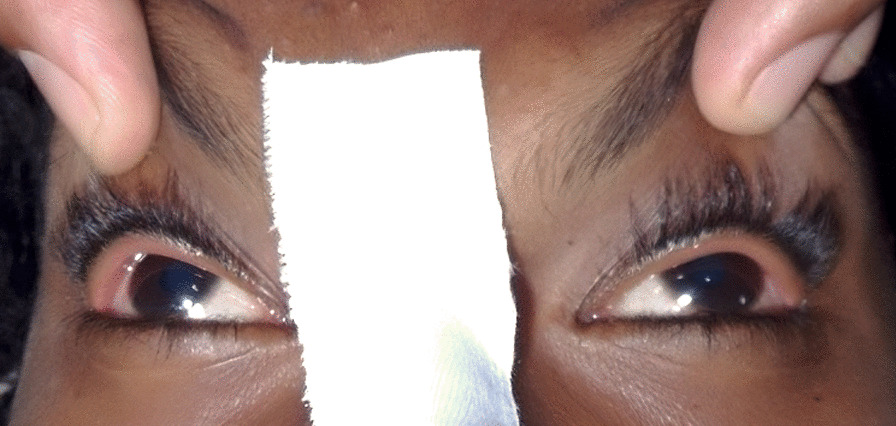
Exotropia seen at presentation to the emergency department

Examination revealed a thin-built (body mass index 17.5 kg m^−2^) girl who was unarousable and unconscious with a Glasgow Coma Scale (GCS) of 8 (eye-opening 2, best verbal response 1, best motor response 5). She had a normal temperature (36 °C). The heart rate was 125 beats per minute, regular in rhythm, blood pressure was 95/50 mmHg, peripheries were warm and nonedematous, and cardiac auscultation was normal. Abdominothoracic respiration was noted at a rate of 15 breaths per minute, with saturation at 97% on air. Lungs were clear and had normal percussion notes. The prominent and unmistakable feature upon opening the eyelids was the divergent strabismus (exotropia), which was previously nonexistent according to the relative (Fig. 1). Her pupils were mydriatic (4 mm) and were sluggishly responsive to light. Ophthalmoscopic examination showed normal retina and optic discs. There were no signs of menigism and no evidence of facial weakness, with intact corneal and vestibular–ocular reflexes. All four limbs were floppy but had brisk reflexes (3+), sustained ankle clonus, and bilaterally positive Babinski response. The only striking abnormality in the abdomen was a dull, palpable pelvic mass, up to the level of the umbilicus, which disappeared upon urinary catheterization. Urine catheterization revealed an immediate output of 2800 ml of clear urine. A thorough examination of the skin did not show any evidence of rashes, animal bites, or fang marks. There was no evidence of injuries to any part of the body, frothing, or lateral tongue bites to suggest recent seizure activity.

**Fig. 2:**
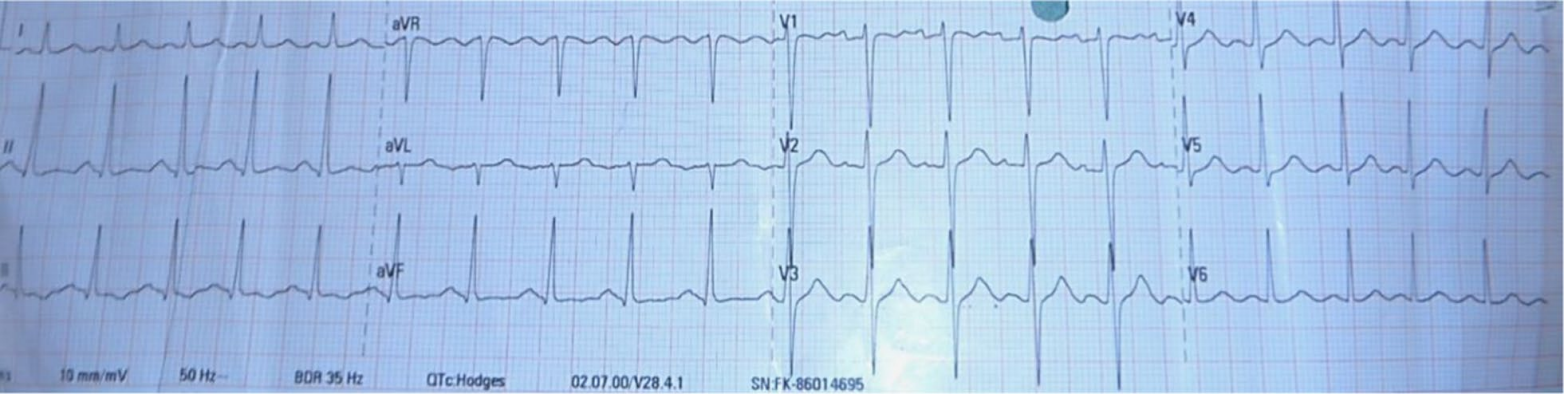
ECG at presentation, showing only tachycardia

**Fig. 3:**
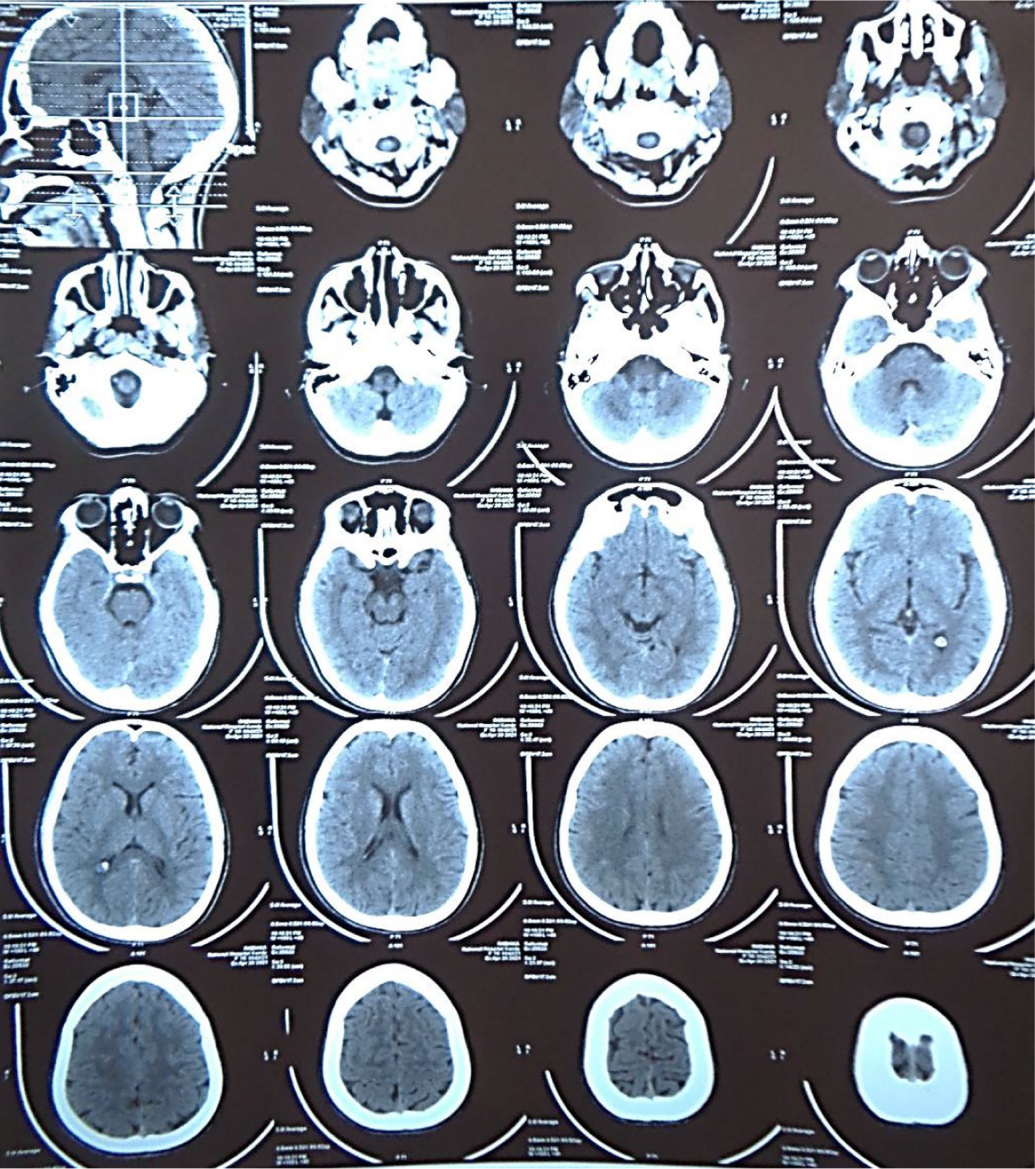
Noncontrast computed tomogram of brain at presentation, reported as normal

An urgent electroencephalogram (ECG) showed no arrhythmia, with a QRS duration of 89 ms, a corrected QT interval of 456 ms, and an R wave/S wave ratio in lead aVR of less than 0.7 (Fig. 2). Bedside venous blood gas showed pH 7.33, p_v_O_2_ of 43.8 mmHg, p_v_CO_2_ of 41.5 mmHg, Sp_v_O_2_ of 75%, HCO_3_^−^ of 22.4 mmol/L, lactate of 0.9 mmol/L, sodium of 139 mmol/L, potassium of 3.5 mmol/L, ionized calcium of 0.9 mmol/L, and glucose of 86 mg/dL. An urgent noncontrast computerized tomography (NCCT) of the brain was reported as normal (Fig. 3). Urine levels of human chorionic gonadotropin were negative, excluding the possibility of an established pregnancy. Whole blood clotting time, a popular tool to screen for snake-venom-related coagulopathy, was unremarkable. A facility for urine toxicology screening was not available, and hence it was not performed.

**Fig. 4:**
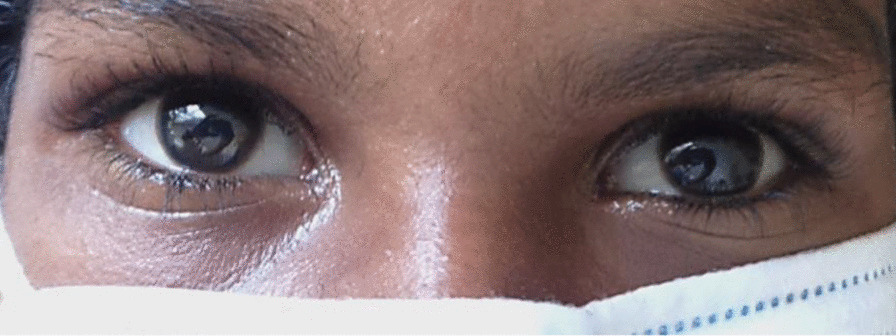
Normal ocular alignment at 36 hours

We tentatively suspected an overdose of amitriptyline as it is commonly prescribed as an analgesic for neuropathic pain in the locality, and the resulting anticholinergic effect would explain most of the clinical examination findings. Activated charcoal (50 g) was administered through a nasogastric tube, and she was transferred immediately to the intensive care unit for continuous ECG and monitoring for neurological deterioration and the development of seizures. Even though there were no sinister ECG abnormalities, the presence of significant neurological deterioration and acidosis made us commence an intravenous 8.4% sodium bicarbonate 100 mg bolus followed by infusion at a rate of 10 mmol/hour. The infusion was withheld after 10 hours as the arterial pH reached 7.45. Intravenous hydration with 10% dextrose at 60 ml/hour and intravenous infusion of 2 g of magnesium sulfate in 100 ml of normal saline were also continued. The results of her investigations are given in Table 1. In 24 hours, her GCS, muscle tone, and heart rate normalized spontaneously. The patient then revealed the information about an impulsive self-overdose of about ten tablets of amitriptyline, which her mother was taking as an analgesic for neuropathic back pain. Subsequently, she was transferred to a general medical ward for further observation. The exotropia completely resolved by 36 hours (Fig. 4). Hyperreflexia and sustained ankle clonus disappeared after 72 hours. She was seen by a specialist psychiatry team and was discharged after 72 hours. An outpatient visit after 1 week did not reveal any residual neurological deficits. Outpatient counseling sessions were continued by the psychiatry team.

**Table 1:** Biochemical parameters at presentation

Parameter	Value
WBC (× 10^3^/mm^3^)	7.88
Hemoglobin (g/dL)	11.4
Platelet count (× 10^3^/mm^3^)	256
C-reactive protein (mg/L)	3.0
Alanine transaminase (U/L)	10.3
Aspartate transaminase (U/L)	15.4
Serum creatinine (umol/L)	54.9
Serum sodium (mmol/L)	143
Serum potassium (mmol/L)	3.9
Serum phosphorus (mmol/L)	1.25
Serum magnesium (mmol/L)	0.8

g - gramme, L - litre, mm^3^ - cubic millimeter, mmol - milimole, WBC - white blood cell, umol - micromole, U - unit

## Discussion

Many prescribed medications, including tricyclic antidepressants, antiepileptic drugs, antipsychotics, antihistamines, antimuscarinic agents, and even the seeds of jimsonweed (*Datura stramonium*), a common Sri Lankan plant, can potentially cause an anticholinergic toxidrome. Unfortunately, we did not have the facilities to confirm the presence of amitriptyline or any of the other drugs in blood or urine. Amitriptyline was the likely cause of poisoning as it is commonly prescribed as an analgesic for neuropathic pain in the locality and also due to the presence of predominant neurological manifestations, which may not be seen in overdoses of all the aforementioned medications.

The toxicological presentation of TCA overdose can be due to anticholinergic manifestations (resulting in urinary retention, xerostomia, mydriasis, tachycardia, ileus, and hyperthermia from impaired sweating) as well as an alpha blockade (resulting in hypotension) [1]. The alpha blockade on fast sodium channels of cardiomyocytes and the cardiac conduction tissue delays the propagation of the depolarization wave, thereby predisposing to dysrhythmias [1]. The prolongation of QRS complexes, PR, and QT intervals are representative of this effect, but they were not seen in our patient. Respiratory depression, too, has been reported from TCA overdose. A mixed acidosis may result from respiratory depression and lactic acidosis from reduced tissue perfusion [1]. Hypokalemia is a less frequently observed metabolic derangement [2]. It is worth noting that an overdose of TCAs will alter the normal pharmacodynamics of the drug as well. The delayed gastric emptying and increased enterohepatic recirculation during TCA overdose will increase the number of unbound TCAs in the bloodstream [3]. Also, the development of metabolic acidosis impairs the protein binding of TCA, further raising the unbound fraction of the drug.

The neurological manifestations of sedation, coma, and seizures are secondary to the TCA’s effect on neuronal sodium channels and due to metabolic acidosis. In addition, the antihistaminic and anticholinergic actions of TCAs also contribute to sedation [4, 5]. Unconsciousness due to TCA overdose usually recovers after 24 hours to 5 days, just as in the reported case [6]. Increased cholinergic transmission in the brainstem is responsible for the altered physiology of horizontal eye movements and dysfunctions of the vestibulocular and oculocephalic reflexes [7]. Mydriasis, cycloplegia, and reduced lacrimation all result from anticholinergic action. Various ocular motor disorders, such as internuclear ophthalmoplegia, total external ophthalmoplegia, or complete loss of brain stem reflexes mimicking brain death, have been previously reported with amitriptyline overdose [6, 8–13]. Several other non-TCA anticholinergic agents (for example, haloperidol and oxybutynin), benzodiazepines, and lithium have also been reported to induce diplopia, esotropia, and eye movement disorders [14, 15]. Amitriptyline may increase the palpebral aperture, giving the appearance of exophthalmos [15]. The pupillary light reflex is often preserved in TCA poisoning, just as in any case of metabolic coma. However, the complete loss of brainstem function from TCA overdose may result in the loss of the light reflex [9]. Transient diminution of corneal, pupillary, and oculocephalic reflexes is considered a poor prognostic feature [9].

As for the management aspects, unstable patients should be promptly resuscitated. Patients with decreased consciousness and respiratory depression require intubation. Gastric decontamination within 1 hour of presentation with lavage or activated charcoal should be cautiously administered in individuals with intact or secure airways [16]. Hypotension should be treated with intravenous fluids and vasopressors if required. ECG monitoring is essential for all. Alkalinization with sodium bicarbonate is recommended in the treatment of dysrhythmias, hypotension, QRS prolongation of more than 100 ms, and should target a serum pH of 7.45–7.55 [16]. There is a place for intravenous magnesium sulfate in refractory dysrhythmia causing hemodynamic instability [16]. However, its use in our patient cannot be rationalized. Benzodiazepines are the mainstay in seizure management, and antiepileptic agents such as phenytoin should be avoided due to interactions with TCAs [16].

Acute-onset, transient, self-limiting exotropia is an uncommon manifestation of amitriptyline overdose and mimics other acute encephalitic and vascular pathologies of the brainstem. Identification of such clinical findings is valuable to the physician providing emergency care, in both resource-rich and resource-deficient countries. This case highlights the importance of being updated on the clinical manifestations of amitriptyline overdose, as it enables one to promptly initiate life-saving therapies upon suspicion.

## Conclusion

The art of clinical examination to gather physical signs and rational interpretation is as valuable and imperative as modern investigational tools in making a diagnosis. The significance of clinical examination is much felt in resource-poor emergency settings, where sophisticated diagnostic tests are either unavailable or not affordable. Identification of an acute or new-onset but short-lasting exotropia in a setting of neurological deterioration warrants looking for the possibility of a tricyclic antidepressant overdose.

## Data Availability

The details of this patient’s reports and images are with the corresponding author.

## Abbreviations

ECG: Electrocardiography
GCS: Glasgow Coma Scale
ms: Millisecond
NCCT: Noncontrast computed tomography
TCA: Tricyclic antidepressant
WBC: White blood cell
